# The Use of Virtual World-Based Cardiac Rehabilitation to Encourage Healthy Lifestyle Choices Among Cardiac Patients: Intervention Development and Pilot Study Protocol

**DOI:** 10.2196/resprot.4285

**Published:** 2015-04-08

**Authors:** LaPrincess C Brewer, Brian Kaihoi, Kathleen K Zarling, Ray W Squires, Randal Thomas, Stephen Kopecky

**Affiliations:** ^1^Mayo Clinic College of MedicineDepartment of MedicineRochester, MNUnited States; ^2^Mayo Clinic College of MedicineDivision of Cardiovascular DiseasesRochester, MNUnited States; ^3^Mayo ClinicCenter for InnovationRochester, MNUnited States; ^4^Mayo Clinic College of MedicineDepartment of NursingRochester, MNUnited States

**Keywords:** cardiac rehabilitation, cardiovascular diseases, eHealth, telemedicine, Internet, health behavior

## Abstract

**Background:**

Despite proven benefits through the secondary prevention of cardiovascular disease (CVD) and reduction of mortality, cardiac rehabilitation (CR) remains underutilized in cardiac patients. Underserved populations most affected by CVD including rural residents, low socioeconomic status patients, and racial/ethnic minorities have the lowest participation rates due to access barriers. Internet-and mobile-based lifestyle interventions have emerged as potential modalities to complement and increase accessibility to CR. An outpatient CR program using virtual world technology may provide an effective alternative to conventional CR by overcoming patient access limitations such as geographics, work schedule constraints, and transportation.

**Objective:**

The objective of this paper is to describe the research protocol of a two-phased, pilot study that will assess the feasibility (Phase 1) and comparative effectiveness (Phase 2) of a virtual world-based (Second Life) CR program as an extension of a conventional CR program in achieving healthy behavioral change among post-acute coronary syndrome (ACS) and post-percutaneous coronary intervention (PCI) patients. We hypothesize that virtual world CR users will improve behaviors (physical activity, diet, and smoking) to a greater degree than conventional CR participants.

**Methods:**

In Phase 1, we will recruit at least 10 patients enrolled in outpatient CR who were recently hospitalized for an ACS (unstable angina, ST-segment elevation myocardial infarction, non-ST-segment elevation myocardial infarction) or who recently underwent elective PCI at Mayo Clinic Hospital, Rochester Campus in Rochester, MN with at least one modifiable, lifestyle risk factor target (sedentary lifestyle, unhealthy diet, and current smoking). Recruited patients will participate in a 12-week, virtual world health education program which will provide feedback on the feasibility, usability, and design of the intervention. During Phase 2, we will conduct a 2-arm, parallel group, single-center, randomized controlled trial (RCT). Patients will be randomized at a 1:1 ratio to adjunct virtual world-based CR with conventional CR or conventional CR only. The primary outcome is a composite including at least one of the following (1) at least 150 minutes of physical activity per week, (2) daily consumption of five or more fruits and vegetables, and (3) smoking cessation. Patients will be assessed at 3, 6, and 12 months.

**Results:**

The Phase 1 feasibility study is currently open for recruitment which will be followed by the Phase 2 RCT. The anticipated completion date for the study is May 2016.

**Conclusions:**

While research on the use of virtual world technology in health programs is in its infancy, it offers unique advantages over current Web-based health interventions including social interactivity and active learning. It also increases accessibility to vulnerable populations who have higher burdens of CVD. This study will yield results on the effectiveness of a virtual world-based CR program as an innovative platform to influence healthy lifestyle behavior and self-efficacy.

## Introduction

### Underutilization of Cardiac Rehabilitation

Coronary artery disease (CAD) has become a pandemic as the leading cause of death worldwide [1]. CAD accounts for over two-thirds of cardiac-related deaths in the US and is associated with significant morbidity [2]. According to the 2014 Heart Disease and Stroke Statistics, an estimated 635,000 Americans suffer from an acute coronary event per year with approximately 300,000 recurrent events in CAD survivors [1]. Targeting behavioral risk factors including poor nutrition, smoking, and physical inactivity as well as conditions such as hypertension, hyperlipidemia, diabetes, and obesity is of utmost importance in order to significantly reduce CAD-associated morbidity and mortality [3-6]. Cardiac rehabilitation (CR) is an essential component of the mainstay therapy for patients following an acute coronary syndrome (ACS), such as acute myocardial infarction, for facilitation of recovery and secondary prevention of further events [7]. Evidence has consistently demonstrated that the comprehensive focus of CR on healthy lifestyle change reduces all-cause and cardiac-related mortality [8-10], and improves exercise capacity, psychosocial well-being, and quality of life [5,11]. However, despite its proven benefits, CR is greatly underutilized, especially among groups that need it most such as ethnic minorities, rural residents, the elderly, and the economically disadvantaged [12,13]. Many of the cited barriers to participation are both personal and systems-related including employment/time conflicts, lack of transportation, geographical accessibility, and financial constraints [12-14].

### Call for Innovation

The American Heart Association (AHA) Presidential Advisory board has recently issued a call for innovative reengineering of the traditional CR model towards approaches to improve adherence and effectiveness in cardiac patients [15]. Novel methods for reaching underserved populations who have the highest prevalence of cardiovascular disease (CVD) are crucially needed to assist in alleviating the burden and disparities within these groups. More Americans are embracing the digital world, with many accessing the Internet for health-related information [16,17]. The use of technology to deliver personalized medicine through mobile and Internet-based interventions has shown promise in improving user’s knowledge, health behaviors, and clinical outcomes [18]. There is recent evidence to suggest the effectiveness of Internet-based CR, termed virtual CR or eRehabilitation through the provision of health promotion programs at the user’s convenience [19-24].

### Study Purpose

Virtual world technology has emerged as a potentially powerful tool for the delivery of lifestyle interventions in the management of chronic diseases including diabetes [25] and obesity [26]. Virtual world environments are unique from Internet-based applications in that they are inherently immersive, engaging, and allow for “real world” interaction though personalized avatars or online personas [27,28]. Users of virtual world are provided with a more synchronous experience allowing for collaborative and experiential learning, skill-building, and “what if” hypothetical scenarios; all core concepts of CR [27,28]. We propose the use of a virtual world interaction as an extension to traditional face-to-face CR as a means for overcoming barriers to CR participation, and for positively impacting cardiac risk factors given its affordances of accessibility, social interactivity, and self-motivation. This virtual world interaction approach could potentially assist in widening access to and participation in CR among the US population as a whole while narrowing the gap in exemplary health outcomes among underserved groups. Our pilot study consists of the following two phases: (1) feasibility and (2) a comparative effectiveness, randomized clinical trial of virtual world based adjunct CR against conventional CR.

## Methods

### Study Setting and Participants

We will recruit patients who were recently hospitalized for an ACS (unstable angina, ST-segment elevation myocardial infarction, non-ST-segment elevation myocardial infarction), or who recently underwent elective percutaneous coronary intervention (PCI) at Mayo Clinic Hospital, Rochester Campus in Rochester, MN with at least one modifiable, lifestyle risk factor target: sedentary lifestyle (<3 hours of physical activity per week), unhealthy diet (consumption of <5 servings of fruits and vegetables daily), and current smoking (>1 year). All patients must have regular high-speed Internet access (either home, work, or community). Patient exclusion criteria will include less than 18 years of age, enrolled in a current CR program, and non-fluent in English. The feasibility research protocol was reviewed and approved by the Mayo Clinic Institutional Review Board and the randomized controlled trial (RCT) will be registered. We include details of the design and methods of both the feasibility study and the proposed RCT as Phases 1 and 2, respectively.

### Phase 1: Feasibility Study

#### Hypothesis

We hypothesize that a virtual world-based CR program can be successfully implemented as an extension of a face-to-face conventional CR program. We plan to evaluate a priori how patients utilize a virtual world-based program for CR and secondary CVD prevention by conducting a 12-week feasibility study. Our goal is to apply the evaluation information obtained from Phase 1 towards the design of a patient-driven and centered virtual world platform prototype (*Destination Rehab*) with high usability, understandability, and credibility.

#### Recruitment

Eligible patients (approximately 10) will be invited to participate from the Mayo Clinic outpatient CR enrollment listings by the study cardiovascular nurse. Evidence supports that at least 5 participants are sufficient to assess usability of Web-based applications [29,30].

#### Intervention

**Figure 1:**
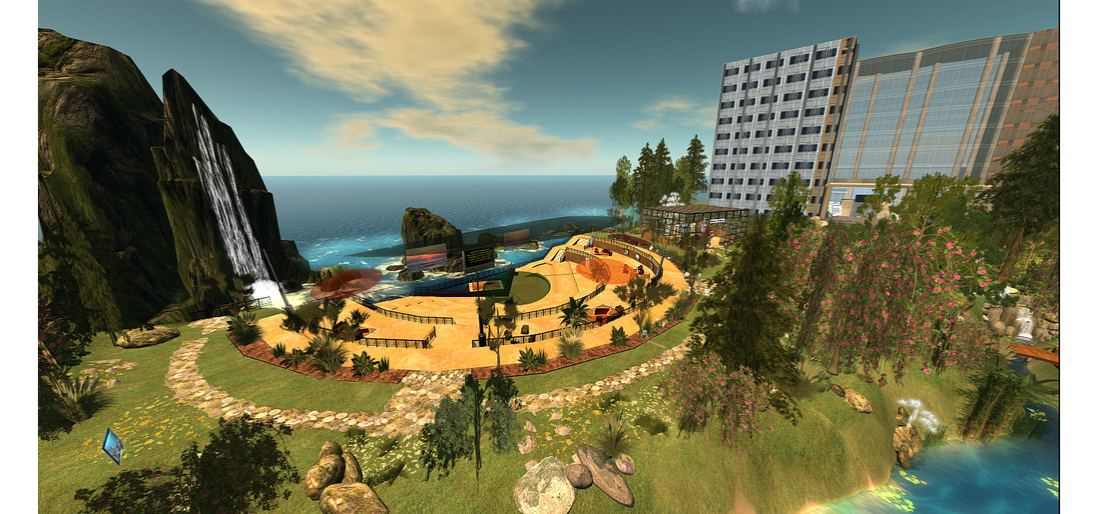
Mayo Clinic conference center virtual world platform in the Second Life application.

**Figure 2:**
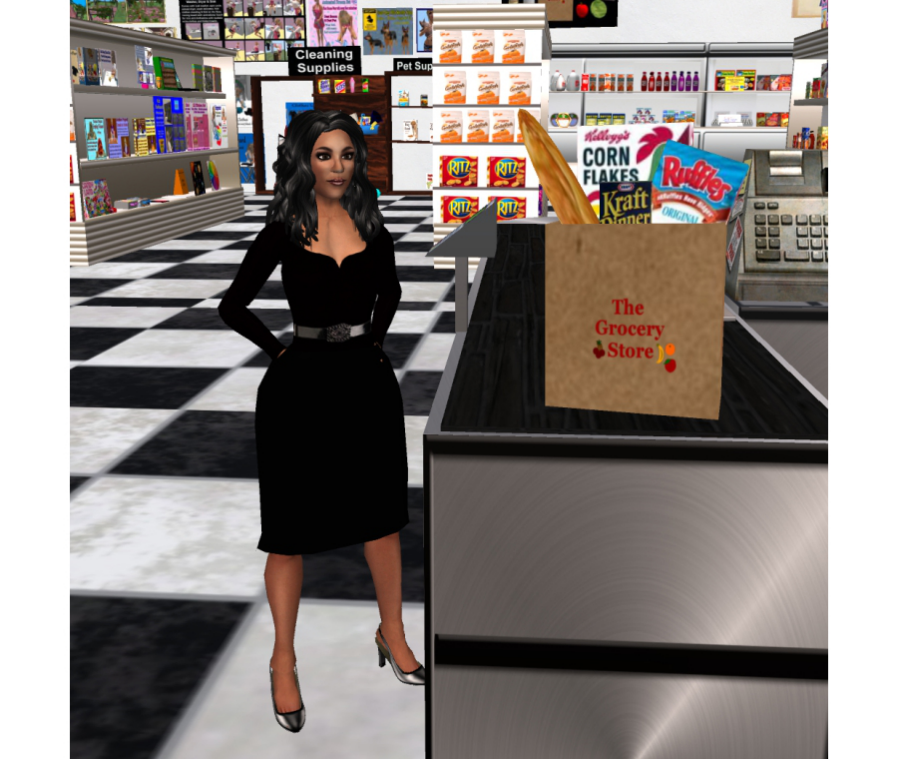
Example of virtual grocery store tour.

**Figure 3:**
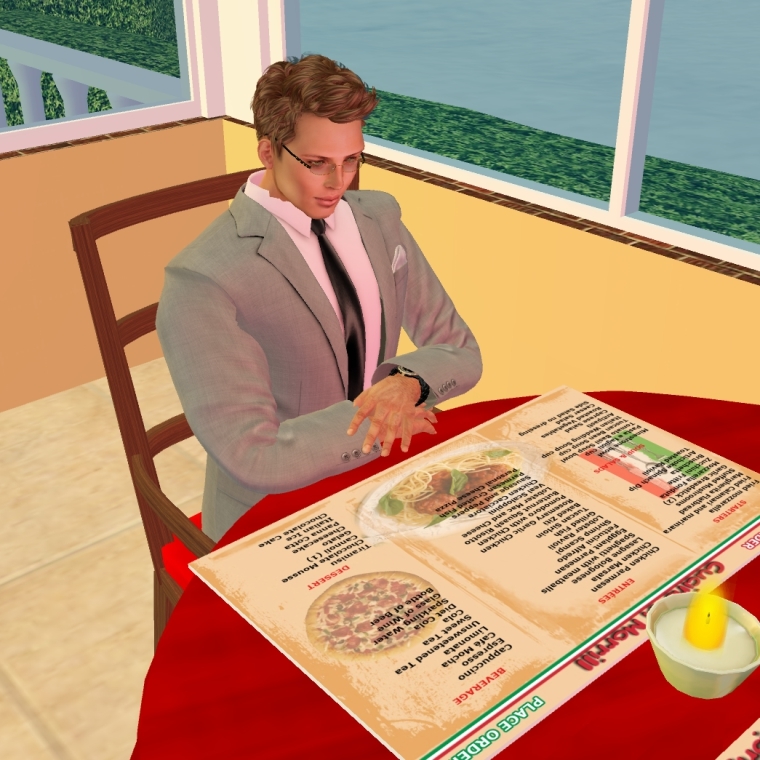
Example of interactive restaurant tour.

**Figure 4:**
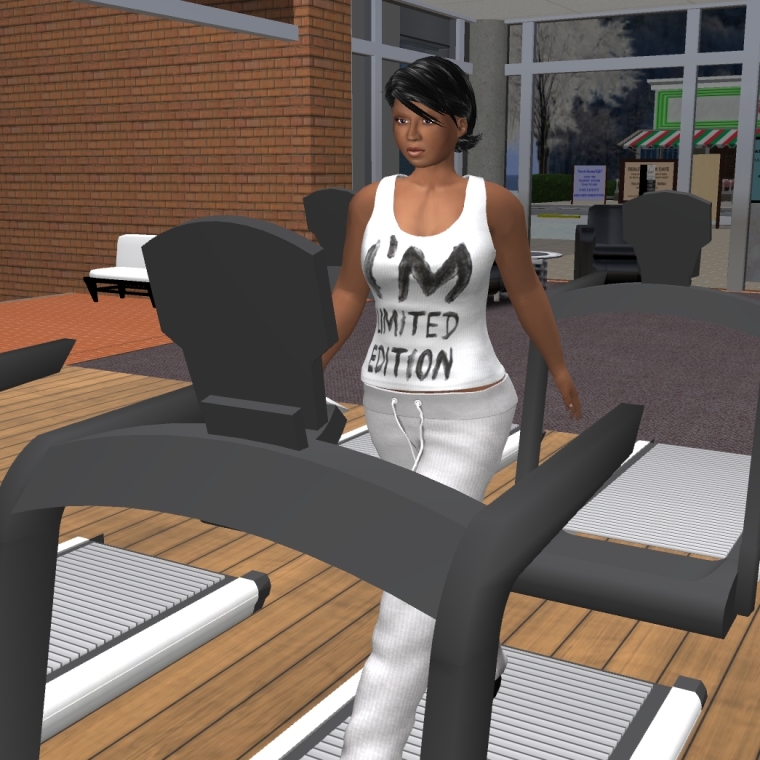
Example of virtual fitness center.

We plan to hold a series of weekly, one-hour sessions over three months on a secure virtual world platform via an established Mayo Clinic infrastructure, Linden Lab’s Second Life, covering relevant cardiovascular health topics including CAD, hypertension, hyperlipidemia, and diabetes (see Textbox 1 and Figure 1) [31]. The sessions will be led by a cardiovascular diseases specialist and a cardiovascular nurse educator both trained in motivational interviewing and the Second Life application. Technical support staff will assist with any virtual world technology technical issues and troubleshooting. We will also hold live “ask-the-expert” group chat sessions on diet and exercise from a dietician and exercise physiologist, respectively. Participants will engage in virtual activities including grocery store and restaurant tours (to discuss healthy food choices, portion control, and nutrition label reading), as well as a variety of fitness activities (see Figures 2-4) [32-34]. Peer discussion forums will be available at all times to the participants. A few of our intervention preparation techniques were adapted from those endorsed by Rosal et al as established processes for virtual world interventions [25]. Participants will receive hands-on training and support including an overview of the virtual world platform, creating a Second Life account (including avatar), and navigation of the *Destination Rehab* prototype. Participants will also be provided with an instructional manual including step-by-step screen shots to support their independent home use. Upon training completion, participants will be provided with a personal laptop for use during the study, complete with required software to access the virtual-world platform and CR program materials, as well as a personal headset with microphone to facilitate communication in virtual world.

**Comparison of virtual world adjunct versus conventional cardiac rehabilitation program curricula.**

Comparison of program curricula

- Virtual world
  - Managing heart disease risk factors (hyperlipidemia, hypertension, diabetes, obesity, and tobacco use)
  - Stress management and relaxation after cardiac events
  - “Ask-the-expert” group chat sessions on nutrition and physical activity with dietician and exercise physiologist
  - Virtual grocery store and interactive restaurant tour with dietician (selecting healthy foods, proper portion sizes, nutrition label reading, etc.)
  - Medications after a cardiac event
  - Sexuality after a cardiac event
  - Peer group “social hour” discussions
- Conventional
  - Managing heart disease risk factors (hyperlipidemia, hypertension, diabetes, obesity, and tobacco use)
  - Stress management and relaxation after cardiac events
  - Dietician nutrition counseling (hints for grocery store shopping, dining out, healthy seasoning, etc.)
  - Cooking demonstration
  - Physical activity counseling with exercise physiologist (personalized exercise program/prescription)
  - Medications after a cardiac event
  - Sexuality after a cardiac event
  - Support groups

#### Measures

We plan to evaluate utility and usability, as well as user satisfaction for each user. Application usage statistics for each participant will be collected to report the frequency and duration of each interaction with the virtual world platform. Furthermore, assessments of participant-to-participant and participant -to-speaker communication during sessions will allow for an appreciation of the participant's sense of immersion and engagement. Feedback on the site usability and utility will be measured by survey and interview consisting of questions from previously validated tools [22,35-38]. The usability questions will include participant impressions of the graphical interface, as well as opinions and attitudes towards the visual appeal (ie, application was designed with the user in mind), content (ie, information was complete), navigation (ie, steps required to complete a task were logical), informational architecture (ie, visual layouts were logical), and interactive features. User satisfaction will be assessed by open response questions to allow participants to provide feedback on likes, dislikes, or concerns about the virtual world platform, and sections to provide recommendations for changes to the program. At Phase 1 completion, study participants will complete a final evaluation survey assessing their willingness to use a similar virtual world platform for CR. Results of this formative evaluation process will inform changes and adjustments to our virtual world program for Phase 2.

### Phase 2: Randomized Controlled Trial

#### Hypothesis

We hypothesize that virtual world CR users will improve behaviors (physical activity, diet, and smoking) to a greater degree than conventional CR participants.

#### Recruitment

Potential participants hospitalized for an ACS or those undergoing elective PCI procedures will be identified from the Division of Cardiovascular Diseases Hospital service census (coronary care unit, general and ischemic ward services, interventional service) by the study team cardiovascular clinical nurse specialist. Each patient will be approached prior to dismissal as part of CR discharge planning during which time they will be provided with pertinent information on the purpose and requirements of the study. Following eligibility screening by the nurse specialist, they may choose to provide written informed consent at the time of recruitment or at another convenient time (ie, baseline visit). Furthermore, eligible patients will be recruited from the Mayo Clinic outpatient CR enrollment listings. Efforts will be implemented to increase priority population (ethnic minorities, rural residents, elderly, and economically disadvantaged) accrual including community-based strategies, clinician-initiated recruitment, advertisements, and medical record review.

#### Baseline Visits

All participants will undergo an outpatient, baseline assessment for collection of sociodemographics, medical history, prescribed medications/adherence [39], smoking status, surveys for heart disease knowledge [40], diet quality [41,42], physical activity patterns [43,44], self-efficacy [41,44,45], social support/influence [46], and physical and mental health-related quality of life [47] utilizing validated instruments after obtainment of written informed consent. Clinical assessments including anthropometrics (height, weight, body mass index, waist circumference, and blood pressure) and laboratory studies (lipid panel, blood glucose, and hemoglobin A1c) will be obtained by standard protocols and/or extracted from participant medical records from their ACS index hospitalization or follow-up medical visits. As a part of the standard clinical CR intake process, all participants will undergo an oxygen consumption exercise treadmill test (ETT) to assess for heart rate and blood pressure responses and exercise capacity. All participants will attend an in-person computer and device training session similar to that carried out during the feasibility study and will receive a personal laptop and headset for use during the study (both control and intervention groups for standardization). Participants will also receive a personal activity tracker (Fitbit^TM^ ) as a tool to accurately assess free-living physical activity [48]. Participants will be asked to wear the activity trackers on a daily basis and to upload their data to a secure webserver at least once weekly. Each participant will receive a binder of education materials (including slide presentations) relevant to their respective assignment.

#### Design and Randomization

The study will consist of a 2-arm, parallel group, single-center RCT. Patients will be randomized at a 1:1 ratio by a computer software-generated list (nQuery advisor) at their baseline outpatient CR visit to adjunct virtual world-based CR with conventional CR or conventional CR only. Randomization will be stratified by block sizes of four.

#### Control Conventional Cardiac Rehabilitation Program

The control group will enroll in a standard center-based CR program, which at Mayo Clinic Rochester consists of 36 sessions over a duration of 12-16 weeks. The comprehensive program includes supervised exercise, cooking demonstrations, didactic lectures, video presentations, group support, and stress management sessions. Participants will have face-to-face access to the medical directors, registered dietician, exercise physiologist, case manager, and stress management specialist. The curriculum course topics and sessions developed according to national standards of care for secondary prevention are outlined in Textbox 1 [49,50].

#### Enhanced Virtual World-Based Intervention

In addition to standard center-based CR, the intervention group will have access to an interactive healthy lifestyle community, *Destination Rehab*, delivered through a virtual world platform on Second Life. The platform will provide specialized educational tools on CVD secondary prevention including information on nutrition, physical activity, smoking, medication adherence, etc. Specific program components will include interactive 3D spaces (ie, grocery store, fitness center, restaurant, virtual library, and human heart tour), and live Mayo Clinic health professional-led education sessions and peer discussion forums (ie, social support groups) (see Figures 1-4) [31-34]. The platform design has the intention of encouraging healthy lifestyle behaviors by participant avatars with the goal of transferring these behaviors to the real world (ie, Proteus effect) [26,28, 51]. The platform features were informed by valuable input from the Mayo Clinic patient and family advisory group (One Voice) at focus groups in January and February 2014, and will be further developed from Phase 1 study results. Weekly education sessions, including slide presentations, will last for 60-90 minutes and allow for interaction between the facilitators and participants through voice chat and text message features (see Multimedia Appendix 1). A summary of the proposed education sessions including key patient-centered elements for promotion of healthy lifestyle change is provided in Textbox 1 [49,50].

#### Measures

The primary outcome is a composite including improvement of at least one of the following 3 cardiac risk factors at baseline, 3, 6, and 12 months (1) physical activity (at least 150 minutes per week), (2) diet (consumption of five or more fruits and vegetables daily), and (3) smoking (complete cessation for baseline smokers, maintained nonsmoking status for baseline nonsmokers). Secondary outcomes will include improvement in all 3 cardiac risk factors, intention and self efficacy to achieving lifestyle change [41,44,45], change in exercise capacity by peak oxygen uptake (VO_2_), change in weight (≥5% weight reduction for patients with baseline BMI >30 kg/m^2^), blood pressure optimization (blood pressure <140/90 mmHg, <130/80 mmHg for diabetics), diabetes control (hemoglobin A1c<7%), hyperlipidemia control (low-density lipoprotein <100 mg/dL), medication adherence [39], social support/influence [46], physical and mental health-related quality of life [47], heart disease knowledge [40], and user evaluation of the virtual world platform (satisfaction, usability, and utility) [22,35-38].

Physical activity will be assessed by the International Physical Activity Questionnaire (IPAQ) which determines physical activity patterns (vigorous-intensity, moderate-intensity, and leisure) over the previous seven days [43]. Furthermore, free-living physical activity data will be obtained from personal activity trackers as number of steps per day [48]. All patients will undergo a symptom-limited ETT with oxygen consumption testing to assess for peak VO_2_ [52,53]. Continuous blood pressure and heart rate measurements, as well as electrocardiograms will be obtained during exercise and recovery periods. A brief dietary recall by a food frequency questionnaire will allow for diet evaluation [42]. Mayo Clinic laboratories will process and analyze all fasting blood specimens including lipid panel (total cholesterol, triglycerides, high density lipoprotein cholesterol, and low density lipoprotein cholesterol), blood glucose, and hemoglobin A1c. Physical examination measures including height, weight, body mass index, waist circumference, and blood pressure will be obtained according to standard guidelines and Mayo Clinic protocols [54-55]. The baseline questionnaires and clinical assessments will be repeated at 3, 6, and 12 months (study completion).

At program completion, we also plan to have semi-structured focus groups to solicit feedback on the intervention and control rehabilitation programs. We anticipate holding at least two sessions (one for each study group) with at least 20 participants per session. We will collect information on participant experiences, attitudes, and beliefs on healthy lifestyle change through open-ended intervention questions developed by the research team. Incentives for participating in the program and for completion of follow-up surveys will be provided to participants (proposed gift certificates and the Mayo Clinic Healthy Heart for Life book).

### Data Management

The data collected from survey materials will be entered and stored electronically on a secure (password-protected) database system (REDCap^TM^) for the duration of the data collection and analysis (estimation one year), and only specified study coordinators/collaborators will have access to the surveys and monitor the data accordingly for research purposes only.

### Sample Size Estimation and Power Calculations

Power analysis for a priori sample size was performed with equivalence testing for two proportions in a randomized design using the program nQuery advisor. Using data on previous research, we estimated that 45% of patients receiving conventional CR, and 74% of patients attending *Destination Rehab* with conventional CR would have at least one correction of a cardiovascular behavioral risk factor at 12 months [29]. Therefore, to discover a clinically-relevant difference of this size between the groups at a 0.05 alpha level with 80% power, we will require 50 participants per group. Assuming a drop-out rate of 10%, we aim to recruit a total of 120 patients for the RCT.

### Statistical Analysis

#### Quantitative Data

For normally distributed variables, simple arithmetic means and standard deviations will be calculated. For categorical variables, frequencies and proportions will be calculated. For clinical endpoints, we plan to calculate changes in measures by comparing differences in change from baseline to follow-up interval. We will include sensitivity analyses with the inclusion of patients with complete data only. Analyses will be performed using commercial software (SAS version 9.2, SAS institute), and a two-tailed value of *P*<.05 as statistically significant.

#### Qualitative Data

Focus group interview data will be recorded, transcribed, and coded by a qualified audio typist and analyst according to a qualitative analysis approach [56]. Descriptive codes by constant comparison methods will then be merged to thematic categories and conceptual frameworks to provide insight to further the enhancement of both CR modalities [57] and barriers towards achieving ideal cardiovascular health. To ensure rigor and accuracy, separate transcription and coding will be conducted by independent analysts from the study team. All data will be processed and analyzed using the NVIVO software package.

## Discussion

### Embracing Virtual Word Technology

Telemedicine and mobile health are rapidly emerging as novel methods of the “virtualization” of healthcare delivery [58]. These technologies may serve as portals to overcome critical barriers to receipt of optimal cardiovascular care among underserved communities including the racial/ethnic minorities, the economically disadvantaged, and elderly who are disproportionately affected by CVD. Virtual world health interventions may offer a solution to this “treatment paradox” by increasing access to those who crucially need evidence-based therapies such as CR [****59]****. Further robust evidence is needed to demonstrate the effectiveness of these interventions in stimulating patient empowerment towards healthy lifestyle behavioral change. Our study will attempt to fulfill this need by assessing the feasibility and clinical effectiveness of CR delivered in a virtual world environment in comparison to standard site-based CR.

### Study Strengths and Limitations

Our study has several strengths mainly given that it is the first study, to our knowledge, assessing the use of virtual technology for CR. Furthermore, it is the only virtual world-based study focused specifically on lifestyle behavior change in patients with ischemic heart disease, the leading cause of morbidity and mortality worldwide [59]. Our study will be conducted at a designated medical center of excellence with inherent patient-centric, comprehensive, and standardized CR services. The development of the program curriculum was guided by the core components and competencies for patients and health professionals as established by the AHA and the American Association of Cardiovascular and Pulmonary Rehabilitation which could facilitate widespread adaptation and insurance reimbursement if deemed effective [49,50]. Our study will randomize study participants to a virtual world intervention as an adjunct to standard CR versus standard CR to ensure receipt of gold standard care by all participants. We will also provide all required hardware, software, and requisite computer training to both study groups. Finally, our study includes both feasibility and comparative analysis components with quantitative and qualitative assessments to ensure scientific rigor and validity.

We recognize that our study has its limitations primarily due to our small sample size, which may limit the generalizability of our results. However, this is justified as this is a feasibility and pilot study using a new method for CR delivery in cardiac patients. Another possible limitation is our provision of a laptop computer to all participants, which may not be practical or sustainable in wide-spread implementation. However, we want to ensure access to all participants and not bias our inclusion criteria by excluding those without a virtual world technology-enabled device.

### Conclusions

It is crucial that we embrace the use of novel technologies to assist cardiac patients in achieving and maintaining healthy behavioral change for secondary prevention. Virtual world technologies may fulfill this need as it has demonstrated effectiveness in improving self-efficacy for chronic disease self management even in socioeconomically disadvantaged populations [25]. We are optimistic that our proposed study of the use of virtual world-based CR will glean informative results on patient acceptability, adaptability, and ultimately empowerment toward de facto cardiovascular risk factor reduction and secondary CVD prevention.

## Multimedia Appendix 1

Virtual world adjunct versus conventional cardiac rehabilitation program for Phase 2 randomized controlled trial.

## Multimedia Appendix 2

CONSORT-EHEALTH checklist V1.6.2 [60].

## Abbreviations

ACS: acute coronary syndrome
AHA: American Heart Association
CAD: coronary artery disease
CR: cardiac rehabilitation
CVD: cardiovascular disease
ETT: exercise treadmill test
IPAQ: International Physical Activity Questionnaire
PCI: percutaneous coronary intervention
RCT: randomized controlled trial
VO_2_: oxygen uptake
