# Supplementary material for: The Use of Virtual World-Based Cardiac Rehabilitation to Encourage Healthy Lifestyle Choices Among Cardiac Patients: Intervention Development and Pilot Study Protocol
Source: JMIR Res Protoc. 2015 Apr 8;4(2):e39. doi: 10.2196/resprot.4285 (PMC4407020; doi:10.2196/resprot.4285)
Supplement: Supplementary file 1 [file resprot_v4i2e39_app1.pdf]

Multimedia Appendix 1

Virtual World Adjunct versus Conventional Cardiac Rehabilitation Program for Phase 2 Randomized Controlled Trial

Virtual World	Conventional
Outpatient baseline visit	

All participants will be provided with the following materials:

- Education materials binder with slide presentations
- Mayo Clinic Heart Healthy for Life book

Baseline assessments will include:

1. **Questionnaire completion**:

   sociodemographics, medical history, prescribed medications/adherence, smoking status, heart disease knowledge, diet quality, physical activity patterns, self-efficacy and quality of life

2. **Anthropometric obtainment**:

   height weight, body mass index, waist circumference, blood pressure

3. **Laboratory studies**:

   lipid panel, hemoglobin A1c

4. **Oxygen consumption exercise treadmill test**

Computer/Device Training Session

All participants will participate in a 2-hour training session and will be provided with the following materials:

- Personal laptop computer
- Personal headset
- Personal activity tracker (Fitbit™)
- Instructional manual binder for virtual world or Internet-based materials

Participants will receive basic training on:	Participants will receive basic training on:
• Laptop, headset and activity tracker hardware	• Laptop, headset and activity tracker hardware
• Connectivity and web browsing	• Connectivity and web browsing
• Procedures for uploading activity tracker data	• Procedures for uploading activity tracker data
• Basics of Second Life® environment including navigation, creation of avatar and communication techniques	• Online curriculum materials on healthy lifestyle change
• Each participant will be assessed on ability to log-in to virtual world platform and basic skills.	

Education Sessions

• All sessions will occur on the Mayo	• All sessions will occur on-site at the

Clinic virtual world platform, *Destination Rehab*

- One-hour session on a weekly basis
- Education delivered by interdisciplinary team including cardiovascular disease specialist, cardiovascular nurse, dietician and exercise physiologist (alternating roles of facilitation and participant chat)
- Slide presentations of key health topics and curriculum (see Table 1) followed by group discussion
- Participant “field trip” tours to the following interactive spaces to learn and practice healthy lifestyle behaviors:
  - Grocery store
  - Fitness center
  - Restaurant
  - Virtual library
  - Human heart tour

Mayo Clinic outpatient cardiac rehabilitation facility

- Face-to-face sessions offered throughout cardiac rehabilitation enrollment schedule
- Education delivered by interdisciplinary team including cardiovascular disease specialist, cardiovascular nurse, dietician and exercise physiologist
- Standard cardiac rehabilitation curriculum (see Table 1) delivered via slide presentations (education binder) and group discussion forums
- Video presentations also available for individual viewing at own convenience
- Prior to or following each session, participants will engage in supervised exercise session at the outpatient center
- All education materials also available on a Mayo Clinic website

- Following each session, participants will engage in a virtual fitness class including Zumba, line dancing, swimming, jogging or rock-climbing
