# Supplementary material for: The Use of Virtual World-Based Cardiac Rehabilitation to Encourage Healthy Lifestyle Choices Among Cardiac Patients: Intervention Development and Pilot Study Protocol
Source: JMIR Res Protoc. 2015 Apr 8;4(2):e39. doi: 10.2196/resprot.4285 (PMC4407020; doi:10.2196/resprot.4285)
Supplement: Supplementary file 2 [file resprot_v4i2e39_app2.pdf]

CONSORT-EHEALTH Checklist V1.6.2 Report

(based on CONSORT-EHEALTH V1.6), available at [http://tinyurl.com/consort-ehealth-v1-6].

4285

Date completed

1/25/2015 1:14:10

by

LaPrincess Brewer

The Use of Virtual World-based Cardiac Rehabilitation to Encourage Healthy Lifestyle Choices Among Cardiac Patients: Intervention Development and Pilot Study Protocol

TITLE

1a-i) Identify the mode of delivery in the title

"We propose the use of a virtual world interaction as an extension to traditional face-to-face cardiac rehabilitation (CR) as a means for overcoming barriers to CR participation and for positively impacting cardiac risk factors given its affordances of accessibility, social interactivity and self-motivation."

1a-ii) Non-web-based components or important co-interventions in title

Not applicable as study aims to assess virtual world technology.

1a-iii) Primary condition or target group in the title

The Use of Virtual World-based Cardiac Rehabilitation to Encourage Healthy Lifestyle Choices Among Cardiac Patients: Intervention Development and Pilot Study Protocol

ABSTRACT

1b-i) Key features/functionalities/components of the intervention and comparator in the METHODS section of the ABSTRACT

"ABSTRACT:
Background: Despite proven benefits through the secondary prevention of cardiovascular disease (CVD) and reduction of mortality, cardiac rehabilitation (CR) remains underutilized in cardiac patients. Underserved populations most affected by CVD including rural residents, low socioeconomic status patients and racial/ethnic minorities, have the lowest participation rates due to access barriers. Internet-and mobile-based lifestyle interventions have emerged as potential modalities to complement and increase accessibility to CR. An outpatient CR program using virtual world technology may provide an effective alternative to conventional CR by overcoming patient access limitations such as geographics, work schedule constraints and transportation.
Objective: The objective of this paper is to describe the research protocol of a two-phased, pilot study that will assess the feasibility (Phase 1) and comparative effectiveness (Phase 2) of a virtual world-based (Second Life®) CR program as an extension of a conventional CR program in achieving healthy behavioral change among post-acute coronary syndrome (ACS) and post-percutaneous coronary intervention (PCI) patients in comparison to a conventional CR program. We hypothesize that virtual world CR users will improve behaviors (physical activity, diet, smoking) to a greater degree than conventional CR participants.
Methods: In Phase 1, we will recruit at least 10 patients enrolled in outpatient CR who were recently hospitalized for an ACS (unstable angina, ST-segment elevation myocardial infarction, non-ST-segment elevation myocardial infarction) or who recently underwent elective PCI at Mayo Clinic Hospital, Rochester Campus in Rochester, MN with at least one modifiable, lifestyle risk factor target (sedentary lifestyle, unhealthy diet and current smoking). Recruited patients will participate in a 12-week, virtual world health education program which will provide feedback on the feasibility, usability and design of the intervention. During Phase 2, we will conduct a 2-arm, parallel group, single-center, randomized controlled trial (RCT). Patients will be randomized at a 1:1 ratio to adjunct virtual world-based CR with conventional CR or conventional CR only. The primary outcome is a composite including at least one of the following: at least 150 minutes per week of physical activity, consumption of five or more fruits and vegetables daily and smoking cessation. Patients will be assessed at 3, 6 and 12 months.
Results: The Phase 1 feasibility study is currently open for recruitment which will be followed by the Phase 2 RCT. The anticipated completion date for the study is May 2016.
Conclusion: Research on the use of virtual world technology in health programs is in its infancy. It offers unique advantages over current web-based health interventions including social interactivity and active learning. It also increases accessibility to vulnerable populations who have higher burdens of CVD. This study will yield results on the effectiveness of a virtual world-based CR program as an innovative platform to influence healthy lifestyle behavior and self-efficacy."

1b-ii) Level of human involvement in the METHODS section of the ABSTRACT

See abstract content in 'subitem 1b-i' section above.

1b-iii) Open vs. closed, web-based (self-assessment) vs. face-to-face assessments in the METHODS section of the ABSTRACT

"We will recruit patients who were recently hospitalized for an ACS (unstable angina, ST-segment elevation myocardial infarction, non-ST-segment elevation myocardial infarction) or who recently underwent elective percutaneous coronary intervention (PCI) at Mayo Clinic Hospital, Rochester Campus in Rochester, MN..."

1b-iv) RESULTS section in abstract must contain use data

"The Phase 1 feasibility study is currently open for recruitment which will be followed by the Phase 2 RCT. In Phase 1, we will recruit at least 10 patients enrolled in outpatient CR. We aim to recruit a total of 120 patients for the RCT."

1b-v) CONCLUSIONS/DISCUSSION in abstract for negative trials

"Our study will assess the feasibility and clinical effectiveness of CR delivered in a virtual world environment in comparison to standard site-based CR."

INTRODUCTION

2a-i) Problem and the type of system/solution

"Despite proven benefits through the secondary prevention of cardiovascular disease (CVD) and reduction of mortality, cardiac rehabilitation (CR) remains underutilized in cardiac patients. Underserved populations most affected by CVD including rural residents, low socioeconomic status patients and racial/ethnic minorities, have the lowest participation rates due to access barriers. Internet-and mobile-based lifestyle interventions have emerged as potential modalities to complement and increase accessibility to CR. An outpatient CR program using virtual world technology may provide an effective alternative to conventional CR by overcoming patient access limitations such as geographics, work schedule constraints and transportation."

2a-ii) Scientific background, rationale: What is known about the (type of) system

See 'subitem 2a-i".

METHODS

3a) CONSORT: Description of trial design (such as parallel, factorial) including allocation ratio

"We hypothesize that virtual world CR users will improve behaviors (physical activity, diet, smoking) to a greater degree than conventional CR participants."

3b) CONSORT: Important changes to methods after trial commencement (such as eligibility criteria), with reasons

Not applicable.

3b-i) Bug fixes, Downtimes, Content Changes

Not applicable.

4a) CONSORT: Eligibility criteria for participants

"We will recruit patients who were recently hospitalized for an ACS (unstable angina, ST-segment elevation myocardial infarction, non-ST-segment elevation myocardial infarction) or who recently underwent elective percutaneous coronary intervention (PCI) at Mayo Clinic Hospital, Rochester Campus in Rochester, MN with at least one modifiable, lifestyle risk factor target: sedentary lifestyle (< 3 hours of physical activity per week), unhealthy diet (consumption of less than 5 servings of fruits and vegetables daily) and current smoking (for more than one year). All patients must have regular high-speed Internet access (either home, work or community). Patient exclusion criteria will include the following: <18 years of age, enrolled in a current CR program and non-fluent in English."

4a-i) Computer / Internet literacy

"Participants will receive hands-on training and support including an overview of the virtual world platform, creating a Second Life® account (including avatar) and navigation of the Destination Rehab prototype. Participants will also be provided with an instructional manual including step-by-step screen shots to support their independent home use. Upon training completion, participants will be provided with a personal laptop for use during the study, complete with required software to access the virtual-world platform and CR program materials as well as a personal headset with microphone to facilitate communication in virtual world."

4a-ii) Open vs. closed, web-based vs. face-to-face assessments:

"Eligible patients (approximately 10) will be invited to participate from the Mayo Clinic outpatient CR enrollment listings by the study cardiovascular nurse. Potential participants hospitalized for an ACS or those undergoing elective PCI procedures will be identified from the Division of Cardiovascular Diseases Hospital service census (coronary care unit, General and Ischemic ward services, Interventional service) by the study team cardiovascular clinical nurse specialist."

4a-iii) Information giving during recruitment

"Each patient will be approached prior to dismissal as part of CR discharge planning during which time they will be provided with pertinent information on the purpose and requirements of the study. Following eligibility screening by the nurse specialist, they may choose to provide written informed consent at the time of recruitment or at another convenient time (i.e., baseline visit)."

4b) CONSORT: Settings and locations where the data were collected

Mayo Clinic Rochester, MN

4b-i) Report if outcomes were (self-)assessed through online questionnaires

A few of our secondary outcomes will be assessed by online surveys.

4b-ii) Report how institutional affiliations are displayed

Not applicable as all participants recruited from Mayo Clinic Rochester, MN.

5) CONSORT: Describe the interventions for each group with sufficient details to allow replication, including how and when they were actually administered

5-i) Mention names, credential, affiliations of the developers, sponsors, and owners

Not applicable.

5-ii) Describe the history/development process

"The platform features were informed by valuable input from the Mayo Clinic patient and family advisory group (One Voice) at focus groups in January and February 2014 and will be further developed from Phase 1 study results. "

5-iii) Revisions and updating

Not applicable as feasibility and pilot study.

5-iv) Quality assurance methods

"..our study includes both feasibility and comparative analysis components with quantitative and qualitative assessment to ensure scientific rigor and validity."

5-v) Ensure replicability by publishing the source code, and/or providing screenshots/screen-capture video, and/or providing flowcharts of the algorithms used

Please see Figures 1-4 and Multimedia Appendix 1.

5-vi) Digital preservation

Please see Figures 1-4 and Multimedia Appendix 1.

5-vii) Access

"In addition to standard center-based CR, the intervention group will have access to an interactive healthy lifestyle community, Destination Rehab, delivered through a virtual world platform on Second Life®. The platform will provide specialized educational tools on CVD secondary prevention including information on nutrition, physical activity, smoking, medication adherence, etc."

"All participants will attend an in-person computer and device training session similar to that carried out during the feasibility study and will receive a personal laptop and headset for use during the study (both control and intervention groups for standardization). "

5-viii) Mode of delivery, features/functionalities/components of the intervention and comparator, and the theoretical framework

Please see "Methods" section for complete description of the Control and Intervention arms.

5-ix) Describe use parameters

"Weekly education sessions, including slide presentations, will last for 60 to 90 minutes and allow for interaction between the facilitators and participants through voice chat and text message features (See Multimedia Appendix 1)."

5-x) Clarify the level of human involvement

"The sessions will be led by a cardiovascular diseases specialist and a cardiovascular nurse educator both trained in motivational interviewing and the Second Life® application. Technical support staff will assist with any virtual world technology technical issues and troubleshooting. "

5-xi) Report any prompts/reminders used

Not applicable.

5-xii) Describe any co-interventions (incl. training/support)

"Participants will receive hands-on training and support including an overview of the virtual world platform, creating a Second Life® account (including avatar) and navigation of the Destination Rehab prototype. Participants will also be provided with an instructional manual including step-by-step screen shots to support their independent home use. Upon training completion, participants will be provided with a personal laptop for use during the study, complete with required software to access the virtual-world platform and CR program materials as well as a personal headset with microphone to facilitate communication in virtual world."

6a) CONSORT: Completely defined pre-specified primary and secondary outcome measures, including how and when they were assessed

"The primary outcome is a composite including improvement of at least one of the following cardiac risk factors at baseline, 3, 6 and 12 months: physical activity (at least 150 minutes per week), diet (consumption of five or more fruits and vegetables daily) and smoking (complete cessation for baseline smokers, maintained nonsmoking status for baseline nonsmokers). Secondary outcomes will include improvement in all three cardiac risk factors, intention and self efficacy to achieving lifestyle change [41, 44-45], change in exercise capacity by peak oxygen uptake (VO2), change in weight (≥5% weight reduction for patients with baseline BMI >30 kg/m2), blood pressure optimization (blood pressure <140/90 mmHg, <130/80 mmHg for diabetics), diabetes control (hemoglobin A1c<7%), hyperlipidemia control (low-density lipoprotein <100 mg/dL), medication adherence [39], social support/influence [46], physical and mental health-related quality of life [47], heart disease knowledge [40] and user evaluation of the virtual world platform (satisfaction, usability and utility) [22, 35-38]."

6a-i) Online questionnaires: describe if they were validated for online use and apply CHERRIES items to describe how the questionnaires were designed/deployed

Note that this is a feasibility and pilot study. All of our questionnaires have been validated online in prior studies.

6a-ii) Describe whether and how “use” (including intensity of use/dosage) was defined/measured/monitored

"Application usage statistics for each participant will be collected to report frequency and duration of each interaction with the virtual world platform."

6a-iii) Describe whether, how, and when qualitative feedback from participants was obtained

"At program completion, we also plan to have semi-structured focus groups to solicit feedback on the intervention and control rehabilitation programs. We anticipate holding at least two sessions (one for each study group) with at least 20 participants per session. We will collect information on participant experiences, attitudes and beliefs on healthy lifestyle change through open-ended intervention questions developed by the research team."

6b) CONSORT: Any changes to trial outcomes after the trial commenced, with reasons

Not applicable as feasibility and pilot study.

7a) CONSORT: How sample size was determined

7a-i) Describe whether and how expected attrition was taken into account when calculating the sample size

"Power analysis for a priori sample size was performed with equivalence testing for two proportions in a randomized design using the program nQuery advisor®. Using data on previous research, we estimated that 45% of patients receiving conventional CR and 74% of patients attending Destination Rehab with conventional CR would have at least one correction of a cardiovascular behavioral risk factor at 12 months [29]. Therefore, to discover a clinically-relevant difference of this size between the groups at a 0.05 alpha level with 80% power, we will require 50 participants per group. Assuming a drop-out rate of 10%, we aim to recruit a total of 120 patients for the RCT. "

7b) CONSORT: When applicable, explanation of any interim analyses and stopping guidelines

Not applicable.

8a) CONSORT: Method used to generate the random allocation sequence

"The study will consist of a 2-arm, parallel group, single-center RCT. Patients will be randomized at a 1:1 ratio by a computer software-generated list (nQuery advisor®) at their baseline outpatient CR visit to adjunct virtual world-based CR with conventional CR or conventional CR only. Randomization will be stratified by block sizes of four."

8b) CONSORT: Type of randomisation; details of any restriction (such as blocking and block size)

See 'subitem 8a'.

9) CONSORT: Mechanism used to implement the random allocation sequence (such as sequentially numbered containers), describing any steps taken to conceal the sequence until interventions were assigned

See 'subitem 8a'.

10) CONSORT: Who generated the random allocation sequence, who enrolled participants, and who assigned participants to interventions

See 'subitem 8a', but this will be generated by the study coordinator, not principal or co-investigators.

11a) CONSORT: Blinding - If done, who was blinded after assignment to interventions (for example, participants, care providers, those assessing outcomes) and how

11a-i) Specify who was blinded, and who wasn’t

Not applicable.

11a-ii) Discuss e.g., whether participants knew which intervention was the “intervention of interest” and which one was the “comparator”

Non-blinded study.

11b) CONSORT: If relevant, description of the similarity of interventions

The virtual world intervention will have similar curricula as standard cardiac rehabilitation, with both of the curriculum course topics and sessions developed according to national standards of care for secondary prevention.

12a) CONSORT: Statistical methods used to compare groups for primary and secondary outcomes

"For normally distributed variables, simple arithmetic means and standard deviations will be calculated. For categorical variables, frequencies and proportions will be calculated. For clinical endpoints, we plan to calculate changes in measures by comparing differences in change from baseline to follow-up interval. We will include sensitivity analyses with inclusion of patients with complete data only. Analyses will be performed using commercial software (SAS, version 9.2; SAS institute) and a two-tailed value of $P < 0.05$ as statistically significant."

12a-i) Imputation techniques to deal with attrition / missing values

Not completed as of yet as this is a feasibility/pilot study.

12b) CONSORT: Methods for additional analyses, such as subgroup analyses and adjusted analyses

Not completed as of yet as this is a feasibility/pilot study.

RESULTS

13a) CONSORT: For each group, the numbers of participants who were randomly assigned, received intended treatment, and were analysed for the primary outcome

Not completed yet as this is a feasibility/pilot study.

13b) CONSORT: For each group, losses and exclusions after randomisation, together with reasons

Not completed yet as this is a feasibility/pilot study.

13b-i) Attrition diagram

Not completed yet as this is a feasibility/pilot study.

14a) CONSORT: Dates defining the periods of recruitment and follow-up

"The Phase 1 feasibility study is currently open for recruitment which will be followed by the Phase 2 RCT. The anticipated completion date for the study is May 2016."

14a-i) Indicate if critical “secular events” fell into the study period

Not applicable.

14b) CONSORT: Why the trial ended or was stopped (early)

Not applicable.

15) CONSORT: A table showing baseline demographic and clinical characteristics for each group

Not completed yet as this is a feasibility/pilot study.

15-i) Report demographics associated with digital divide issues

Not completed yet as this is a feasibility/pilot study.

16a) CONSORT: For each group, number of participants (denominator) included in each analysis and whether the analysis was by original assigned groups

16-i) Report multiple “denominators” and provide definitions

Not completed yet as this is a feasibility/pilot study.

16-ii) Primary analysis should be intent-to-treat

Not completed yet as this is a feasibility/pilot study.

17a) CONSORT: For each primary and secondary outcome, results for each group, and the estimated effect size and its precision (such as 95% confidence interval)

Not completed yet as this is a feasibility/pilot study.

17a-i) Presentation of process outcomes such as metrics of use and intensity of use

Not completed yet as this is a feasibility/pilot study.

17b) CONSORT: For binary outcomes, presentation of both absolute and relative effect sizes is recommended

Not completed yet as this is a feasibility/pilot study.

18) CONSORT: Results of any other analyses performed, including subgroup analyses and adjusted analyses, distinguishing pre-specified from exploratory

Not completed yet as this is a feasibility/pilot study.

18-i) Subgroup analysis of comparing only users

Not completed yet as this is a feasibility/pilot study.

19) CONSORT: All important harms or unintended effects in each group

Not completed yet as this is a feasibility/pilot study.

19-i) Include privacy breaches, technical problems

Not applicable.

19-ii) Include qualitative feedback from participants or observations from staff/researchers

Not completed yet as this is a feasibility/pilot study.

DISCUSSION

20) CONSORT: Trial limitations, addressing sources of potential bias, imprecision, multiplicity of analyses

20-i) Typical limitations in ehealth trials

"We recognize that our study has its limitations primarily due to our small sample size which may limit the generalizability of our results. However, this is justified as this is a feasibility and pilot study using a new method for CR delivery in cardiac patients. Another possible limitation is our provision of a laptop computer to all participants which may not be practical or sustainable in wide-spread implementation. However, we want to ensure access to all participants and not bias our inclusion criteria by excluding those without a virtual world technology-enabled device."

21) CONSORT: Generalisability (external validity, applicability) of the trial findings

21-i) Generalizability to other populations

"Furthermore, our study will be conducted at a designated medical center of excellence with inherent patient-centric, comprehensive and standardized CR services. The development of the program curriculum was guided by the core components and competencies for patients and health professionals as established by the AHA and the American Association of Cardiovascular and Pulmonary Rehabilitation (AACVPR) which could facilitate widespread adaptation and insurance reimbursement if deemed effective [49-50]."

21-ii) Discuss if there were elements in the RCT that would be different in a routine application setting

See 'subitem 20-i".

22) CONSORT: Interpretation consistent with results, balancing benefits and harms, and considering other relevant evidence

22-i) Restate study questions and summarize the answers suggested by the data, starting with primary outcomes and process outcomes (use)

Not completed yet as this is a feasibility/pilot study.

22-ii) Highlight unanswered new questions, suggest future research

"We are optimistic that our proposed study of the use of virtual world-based CR will glean informative results on patient acceptability, adaptability and ultimately empowerment toward de facto cardiovascular risk factor reduction and secondary CVD prevention."

Other information

23) CONSORT: Registration number and name of trial registry

"The feasibility research protocol was reviewed and approved by the Mayo Clinic Institutional Review Board and the randomized controlled trial (RCT) will be registered on ClinicalTrials.gov."

24) CONSORT: Where the full trial protocol can be accessed, if available

Not applicable.

25) CONSORT: Sources of funding and other support (such as supply of drugs), role of funders

This study is funded by a grant from the Mayo Clinic Center for Clinical and Translational Science.

X26-i) Comment on ethics committee approval

"The feasibility research protocol was reviewed and approved by the Mayo Clinic Institutional Review Board and the randomized controlled trial (RCT) will be registered on ClinicalTrials.gov. "

x26-ii) Outline informed consent procedures

"Potential participants hospitalized for an ACS or those undergoing elective PCI procedures will be identified from the Division of Cardiovascular Diseases Hospital service census (coronary care unit, General and Ischemic ward services, Interventional service) by the study team cardiovascular clinical nurse specialist. Each patient will be approached prior to dismissal as part of CR discharge planning during which time they will be provided with pertinent information on the purpose and requirements of the study. Following eligibility screening by the nurse specialist, they may choose to provide written informed consent at the time of recruitment or at another convenient time (i.e., baseline visit)."

X26-iii) Safety and security procedures

"The data collected from survey materials will be entered and stored electronically on a secure (password-protected) database system (REDCapTM) for the duration of the data collection and analysis (estimation one year) and only specified study coordinators/collaborators will have access to the surveys and monitor the data accordingly for research purposes only."

X27-i) State the relation of the study team towards the system being evaluated

None declared.
